# Innovative strategies for salinity stress management in Mango nursery plants: *Agrobacterium fabrum* and Glycine betaine approach

**DOI:** 10.1186/s12870-025-07947-z

**Published:** 2025-12-19

**Authors:** Arwa A. AL-Huqail, Muhammad Ikram, Asif Minhas, Muhammad Mehran, Hasseb Ur Rehman, Maged M. Alharbi, Esawy Mahmoud, Farahat S. Moghanm, Adel M. Ghoneim

**Affiliations:** 1 Department of Biology, College of Science, Princess Nourah bint Abdulrahman University, P.O. Box 84428, Riyadh, 11671 Saudi Arabia; 2 Institute of Agronomy, Faculty of Agricultural Science’s and Technology Bahauddin Zakariya University Multan, Multan, Pakistan; 3 Institute of Soil Science, Faculty of Agricultural Science’s and Technology Bahauddin Zakariya University Multan, Multan, Pakistan; 4 Ministry of Environment, Water and Agriculture, Saudi Irrigation Organization (SIO), Riyadh, 14511 Saudi Arabia; 5 Soil and Water Department, Faculty of Agriculture, Tanta University, Tanta, Egypt; 6 Soil and Water Department, Faculty of Agriculture, Kafr Elsheikh University, Kafr El-Sheikh, 33516 Egypt; 7 Agricultural Research Center, Field Crops Research Institute, Giza, 12112 Egypt

**Keywords:** Salt stress, A. fabrum, Secondary metabolites, Oxidative stress, Mangifera indica

## Abstract

This study investigated the impact of *Agrobacterium fabrum* (A. fabrum) and glycine betaine (GB) on mango seedling performance under saline and non-saline conditions using a randomized complete block design with three replications. Salinity stress was imposed based on electrical conductivity (EC), with two levels: 2.77 dS m^− 1^ (non-saline) and 6.56 dS m^− 1^ (saline). Application of *A. fabrum* in combination with 0.4% GB significantly improved seedling growth and physiology under salt stress. Agronomic traits, including scion and rootstock height, diameter, and biomass (fresh and dry weights), were markedly enhanced, with increases ranged from 18.34 to 79.47%. Physiological parameters such as chlorophyll content, relative water content, photosynthetic rate, stomatal conductance, and transpiration rate were also improved by about 20.04–88.06%. Mechanistically, these improvements suggest that *A. fabrum* likely enhanced nutrient uptake and root vigor, while GB functioned as an osmoprotectant, stabilizing cellular structures and maintaining water balance. Their synergistic action effectively mitigated salt-induced oxidative and osmotic stress, thereby promoting overall mango seedling resilience.

## Introduction

 Salinity stress is a formidable challenge in modern agriculture, negatively affecting crop growth, productivity, and quality in salt-prone regions. Globally, soil salinization impacts approximately 1100 million hectares (Mha), accounting for nearly 7% of the Earth’s land surface [1–3]. The problem arises from both natural geochemical processes and anthropogenic activities. Primary salinization occurs through atmospheric deposition, sea level rise, and seawater intrusion, while secondary salinization is exacerbated by poor irrigation practices, excessive fertilizer applications, and intensified agricultural activities [4–6]. These conditions severely restrict crop cultivation, particularly in arid and semi-arid regions. Developing strategies to improve plant salinity tolerance remains a crucial step for sustaining agricultural production under these challenging environments [7].

Mango (*Mangifera indica L.*), a fruit crop of great economic and nutritional value, is highly sensitive to salinity stress. Mango trees accumulate sodium (Na⁺) in leaves at a rate 2.5–3.0 times higher than many other plant species, which disrupts ion homeostasis, reduces photosynthetic efficiency, and accelerates leaf necrosis [8]. As a result, salinity often causes leaf scorching, curling, stunted growth, premature defoliation, and even mortality [9]. Physiological processes such as stomatal conductance, photosynthesis, and water potential are also adversely affected, ultimately reducing growth and yield [10–12].

Glycine betaine (GB), a quaternary ammonium compound, is one of the most important compatible osmolytes involved in osmoregulation, helping to regulate cellular water potential and protect against abiotic stresses like salinity and drought. Accumulating primarily in chloroplasts, GB stabilizes thylakoid membranes, preserves enzyme activity, and scavenges reactive oxygen species (ROS) under salt stress [13–15]. Exogenous application of GB has been shown to enhance photosynthetic efficiency, maintain water balance, and protect cellular structures, thereby improving plant performance in saline conditions [16]– [17].

In parallel, beneficial microorganisms such as plant growth-promoting rhizobacteria (PGPR) have emerged as sustainable tools to enhance crop resilience under abiotic stresses [18]– [19]. *Agrobacterium fabrum*, although widely studied for genetic transformation, has also demonstrated potential to improve plant stress tolerance by modulating phytohormones, enhancing nutrient uptake, and regulating stress-related gene expression. While direct studies on mango are limited, related plant growth-promoting rhizobacteria (PGPR) such as *Azospirillum brasilense*, *Bacillus subtilis*, and *Pseudomonas fluorescens* have improved salt tolerance in several crops by enhancing antioxidant activity, osmolyte accumulation, and Na⁺/K⁺ balance [20]. In mango, symbionts like arbuscular *mycorrhizal fungi* (AMF) and *Azotobacter* have been reported to enhance water use efficiency and nutrient uptake under salt stress [21]. These findings highlight the promise of *A. fabrum* as a PGPR candidate for salinity stress mitigation in mango.

Despite these advances, the combined use of PGPR such as *A. fabrum* with osmoprotectants like GB in mango has not yet been systematically evaluated. We hypothesize that their integration would provide synergistic benefits: *A. fabrum* enhancing nutrient acquisition and hormonal balance, while GB stabilizes cellular structures and osmotic homeostasis.

Therefore, this study aims to investigate the individual and interactive effects of *A. fabrum* and GB on growth and physiological responses of mango seedlings under saline and non-saline conditions. By addressing this knowledge gap, our research introduces a novel and sustainable strategy for enhancing mango resilience to salinity stress, with potential applications for fruit production in salt-affected regions [22–24].

## Materials and methods

### Plant materials and growth conditions

A jointly pot culture experiment was conducted in the wire-house facility of the Faculty of Agricultural Science and Technology (30°15′49″N, 71°30′35″E) . The experiment used a single mango (*Mangifera indica L.*) cultivar, Sufaid Chaunsa, known for its fruit quality and commercial importance. Eighteen-month-old grafted seedlings (± 5 days) were obtained from the Mango Research Institute, Multan, Pakistan. All grafts were made onto *Desi* rootstock, a vigorous, locally adapted rootstock, raised from a true-to-type seed source to ensure genetic uniformity. Uniform seedlings were selected based on scion–rootstock diameter and plant height.

### Salinity development

Salinity was maintained at two levels, 2.77 dS m^− 1^ (non-saline) and 6.56 dS m^− 1^ (saline), by adding analytical-grade of NaCl to the soil mixture. Electrical conductivity (EC) was monitored using a digital conductivity meter at regular intervals to ensure treatment stability [25].

### Experimental design and treatments

The experiment was conducted using a two-factor completely randomized design (CRD) with three replications. Four treatments were applied: control (tap water only), *A. fabrum* inoculation, 0.4% Glycine betaine (GB) as foliar application, and a combined treatment of 0.4% GB + *A. fabrum*. Each treatment was tested under two defined salinity conditions. Each treatment × salinity combination included five plants per replication, resulting in 15 plants per treatment and a total of 120 plants in the entire experiment (4 treatments × 2 salinity levels × 5 plants × 3 replications).

### Sample processing and treatment application

Following a 120-day treatment period, selected based on the typical developmental cycle of mango seedlings and supported by preliminary observations, the plants were harvested [26]– [27]. At harvest, seedlings were separated into leaves, roots, rootstock, and scion, rinsed with distilled water, patted dry, and air-dried at ambient temperature. Fresh weights of each component were recorded using a top-loading balance, plant height was measured with a measuring tape, and stem diameter with a Vernier caliper. Dry weights were obtained by oven-drying samples at 80 °C for 72 h until constant weight. The dried samples were then used for chemical analyses, which were performed at the Soil and Plant Physiology Laboratory, Department of Agronomy, Bahauddin Zakariya University, Multan, Pakistan following standard protocols described in [13]. For biological treatments, the *Agrobacterium fabrum* strain (ATCC 23308) was cultured in LB broth, and the inoculum was standardized to ~ 10⁸ CFU/mL at Department of Biology, College of Science Princess Nourah bint Abdulrahman University, Saudi Arabia. Each seedling received 50 mL of inoculum as a soil drench at transplanting, and the treatment was repeated once more after 30 days to ensure successful root colonization. For the GB treatment, a 0.4% solution was prepared following the method of [28] and applied as a foliar spray at fortnightly intervals, beginning from two weeks after transplanting and continued throughout the 120-day period. Sprays were applied with a hand sprayer until leaf surfaces were thoroughly wetted. All treatments were thus maintained for 120 days, corresponding to the developmental cycle of mango seedlings (Table 1).

**Table 1:** Chemical properties of the soil used in the experiment

Soil properties	2.77 dS m^− 1^	6.56 dS m^− 1^	References
pH	8.17	8.34	[29]
EC (dS m^− 1^)	2.77	6.56	[30]
Organic matter (%)	0.69	0.44	[31]
N (%)	0.032	0.021	[32]
P (mg kg^− 1^)	5.34	3.56	[33]
K (mg kg^− 1^)	120	93	[34]
Texture	Sandy loam	Sandy loam	[35]

### Electrolyte leakage (EL)

Electrolyte leakage was determined as an indicator of cell membrane damage and stress. This formula reflects the increase in conductivity due to cell membrane damage caused by stress [36].

Electrolyte leakage was calculated as the percentage of conductivity increases:

$$\:\mathrm{E}\mathrm{L}\:\left(\mathrm{\%}\right)=\frac{\left(\mathrm{C}2-\mathrm{C}1\text{}\right)}{{\mathrm{C}}_{0}}\:\times\:100$$

Where C₁ is the initial conductivity, C₂ is the final conductivity after 24 h, and C₀ is the conductivity of deionized water.

### Relative water content (RWC)

Leaf samples were collected, and their fresh weights were recorded. Then, they were soaked in distilled water for a few hours, blotted dry, and re-weighed to obtain turgid weight. Finally, the samples were dried in an oven and weighed again to determine dry weight.

RWC was calculated using the formula:

$$\:\mathrm{R}\mathrm{W}\mathrm{C}\left(\mathrm{\%}\right)=\frac{(\mathrm{F}\mathrm{r}\mathrm{e}\mathrm{s}\mathrm{h}\:\mathrm{W}\mathrm{e}\mathrm{i}\mathrm{g}\mathrm{h}\mathrm{t}\:-\:\mathrm{D}\mathrm{r}\mathrm{y}\:\mathrm{W}\mathrm{e}\mathrm{i}\mathrm{g}\mathrm{h}\mathrm{t})\:}{(\mathrm{T}\mathrm{u}\mathrm{r}\mathrm{g}\mathrm{i}\mathrm{d}\:\mathrm{W}\mathrm{e}\mathrm{i}\mathrm{g}\mathrm{h}\mathrm{t}\:-\:\mathrm{D}\mathrm{r}\mathrm{y}\:\mathrm{W}\mathrm{e}\mathrm{i}\mathrm{g}\mathrm{h}\mathrm{t})}\times\:100$$

### Gas-exchange parameters

Gas-exchange parameters, including photosynthetic rate, stomatal conductance, internal CO₂ concentration, and transpiration, were measured between 9:00 and 11:00 am using a portable photosynthesis system (LI-6200, LI-COR Inc., Lincoln, NE, USA), following methods adapted for mango physiology [37–40]. Measurements were conducted over several consecutive days and randomized across treatments to minimize diurnal and operator bias.

### Statistical analysis

Statistical analyses were carried out using Statistix 8.1 software. Prior to analysis, data were tested for normality (Shapiro–Wilk test) and homogeneity of variance (Levene’s test). A two-way ANOVA was used to assess the effects of salinity, treatments, and their interaction. When significant differences were detected (*p* ≤ 0.05), Tukey’s Honest Significant Difference (HSD) test was employed for mean separation. Results are presented as mean ± standard error (SE). Graphs were created using OriginPro 2024 for effective data visualization.

## Results

### Agronomic characteristics of Mango seedlings

#### Scion height

**Fig. 1:**
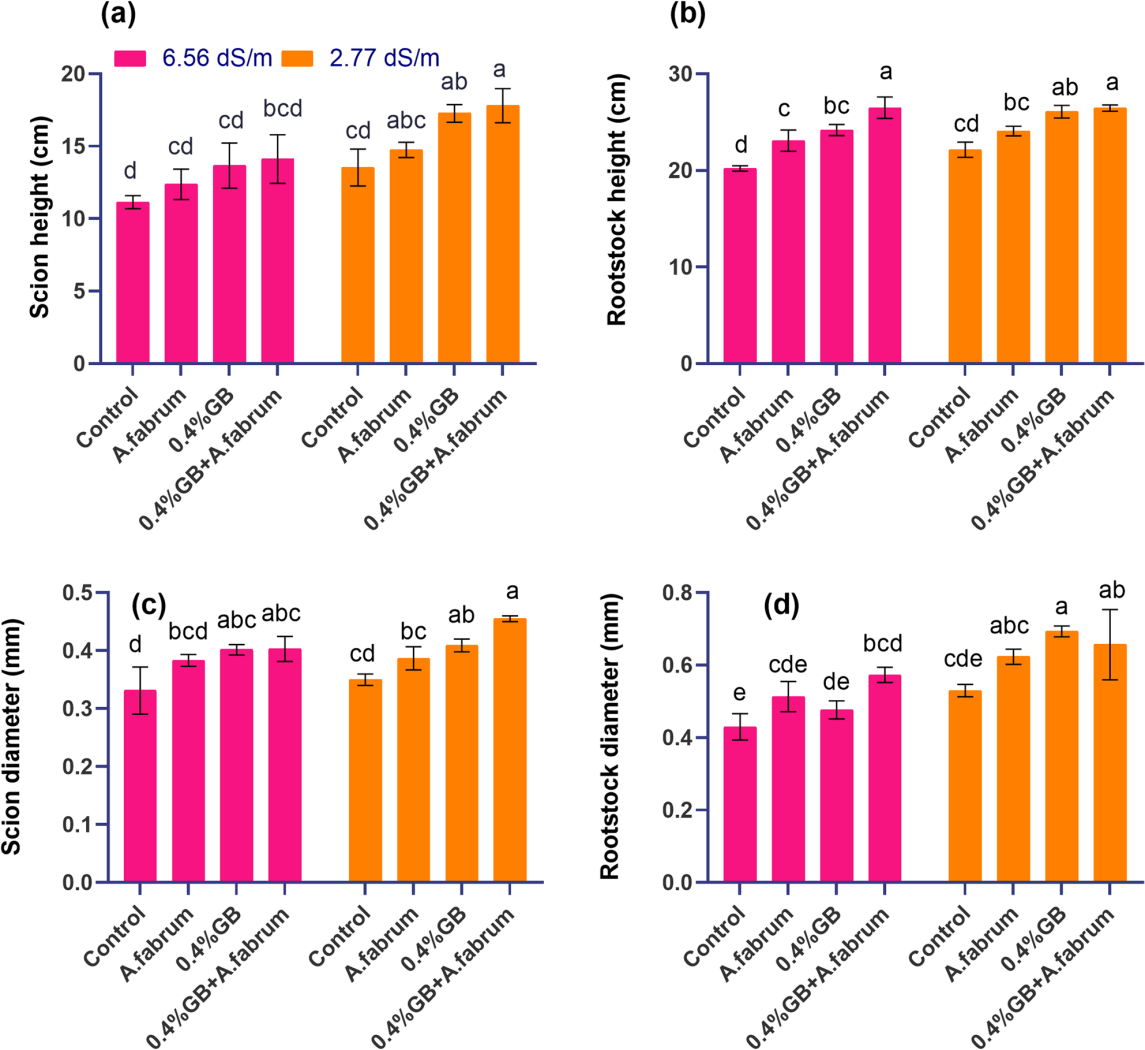
Effect of applied treatments on scion height (**a**), rootstock height (**b**), scion diameter (**c**) and rootstock diameter (**d**) of mango seedlings under saline and non-saline growth conditions. The bars are showing the mean (*n* = 3) and different letters are showing the statistical differences of Tukey’s HSD test among the treatments at *p* ≤ 0.05

The study investigated the effect of salinity stress on the scion height (cm) of mango seedlings under various treatments. At a salinity level of 6.56 dS m^− 1^, the control group showed a mean scion height of 11.14 cm. Treatment with *A. fabrum* resulted in a mean scion height of 12.37 cm, representing an 11.04% increase compared to the control. The application of 0.4% GB led to a further increase in scion height, with a mean of 13.67 cm, corresponding to a 22.64% increase over the control. Combining 0.4% GB with *A. fabrum* resulted in the highest mean scion height of 14.12 cm, indicating a 26.71% increase compared to the control. At a lower salinity level of 2.77 dS m^− 1^, the control group exhibited a mean scion height of 13.54 cm. Treatment with *A. fabrum* led to a slight increase in scion height to 14.75 cm, representing an 8.95% increase compared to the control. The introduction of 0.4%GB resulted in a significant boost in scion height, with a mean of 17.27 cm, indicating 27.54% increases over the control. Similarly, combining 0.4%GB with *A. fabrum* yielded the highest mean scion height of 17.80 cm, indicating a substantial 31.48% increase compared to the control (Fig. 1a).

#### Rootstock height (cm)

At a salinity stress level of 6.56 dS m^− 1^, the control group displayed a mean rootstock height of 20.22 cm. Treatment with *A. fabrum* resulted in a mean height of 23.09 cm, indicating a 14.19% increase compared to the control. Introducing 0.4%GB led to further growth, with a mean height of 24.19 cm, representing a 19.64% increase over the control. Combining 0.4%GB with *A. fabrum* resulted in the highest mean rootstock height of 26.49 cm, demonstrating a substantial 31.02% increase compared to the control. At a lower salinity stress level of 2.77 dS m^− 1^, the control group exhibited a mean rootstock height of 22.15 cm. Treatment with *A. fabrum* led to a slight increase in height to 24.08 cm, indicating an 8.74% increase compared to the control. The introduction of 0.4%GB resulted in a significant boost in rootstock height, with a mean of 26.08 cm, indicating a 17.77% increase over the control. Similarly, combining 0.4%GB with *A. fabrum* yielded a mean rootstock height of 26.45 cm, representing a substantial 19.44% increases compared to the control (Fig. 1b).

#### Scion diameter

At a salinity stress level of 6.56 dS m^− 1^, the control group displayed a mean scion diameter of 0.33 cm. Treatment with *A. fabrum* resulted in a mean diameter of 0.38 cm, indicating a 15.66% increase compared to the control. Introducing 0.4%GB led to further growth, with a mean diameter of 0.40 cm, representing a 21.19% increase over the control. Interestingly, combining 0.4%GB with *A. fabrum* also yielded a mean scion diameter of 0.40 cm, demonstrating a similar 21.59% increase compared to the control. At a lower salinity stress level of 2.77 dS m^− 1^, the control group exhibited a mean scion diameter of 0.35 cm. Treatment with *A. fabrum* led to a slight increase in diameter to 0.39 cm, indicating a 10.57% increase compared to the control. The application of 0.4%GB resulted in a significant boost in scion diameter, with a mean of 0.41 cm, indicating 16.95% increases over the control. Remarkably, combining 0.4%GB with *A. fabrum* resulted in the highest mean scion diameter of 0.46 cm, demonstrating a substantial 30.00% increases compared to the control (Fig. 1c).

#### Rootstock diameter

At a salinity stress level of 6.56 dS m^− 1^, the control group displayed a mean rootstock diameter of 0.43 cm. Treatment with *A. fabrum* resulted in a mean diameter of 0.51 cm, indicating 18.80% increases compared to the control. Introducing 0.4%GB led to a slightly lower mean diameter of 0.48 cm, representing a 10.68% increase over the control. However, combining 0.4%GB with *A. fabrum* resulted in the highest mean rootstock diameter of 0.57 cm, demonstrating a substantial 33.12% increases compared to the control. At a lower salinity stress level of 2.77 dS m^− 1^, the control group exhibited a mean rootstock diameter of 0.53 cm. Treatment with *A. fabrum* led to a mean diameter of 0.62 cm, indicating 16.94% increases compared to the control treatment. The introduction of 0.4%GB resulted in a significant boost in rootstock diameter, with a mean of 0.70 cm, indicating 30.85% increases over the control. Remarkably, combining 0.4%GB with *A. fabrum* resulted in a mean rootstock diameter of 0.66 cm, demonstrating a substantial 23.40% increase compared to the control (Fig. 1d). Figure 1 shows the effect of treatments on scion and rootstock height. Additionally, a detailed analysis of the interaction between salinity (EC) and the treatments is included. This interaction explains how the combination of *A. fabrum* and Glycine betaine (GB) mitigated salt stress under both saline and non-saline conditions, leading to enhanced growth of mango seedlings.

#### Scion fresh weight

**Fig. 2:**
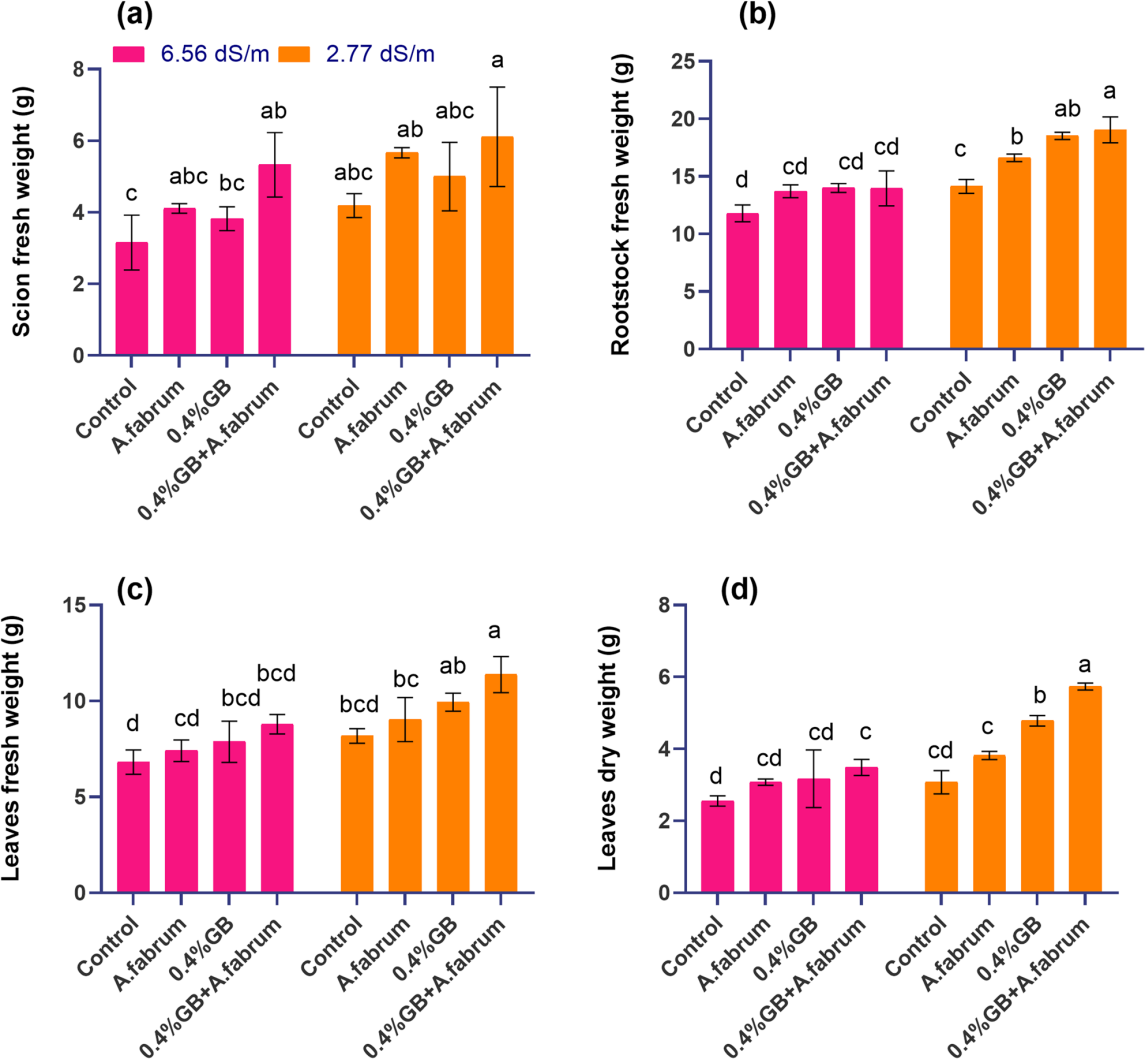
Effect of applied treatments on scion fresh weight (**a**), rootstock fresh weight (**b**), leaf fresh weight (**c**) and leaf dry weight (**d**) of mango seedlings under saline and non-saline growth conditions. The bars are showing the mean (*n* = 3) and different letters are showing the statistical differences of Tukey’s HSD test among the treatments at *p* ≤ 0.05

At a salinity stress level of 6.56 dS m^− 1^, the control group displayed a mean scion fresh weight of 3.16 g. Treatment with *A. fabrum* resulted in a mean fresh weight of 4.11 g, indicating 30.26% increases compared to the control. Introducing 0.4%GB led to a slightly lower mean fresh weight of 3.82 g, representing 21.17% increases over the control treatment. However, combining 0.4%GB with *A. fabrum* resulted in the highest mean scion fresh weight of 5.33 g, demonstrating a substantial 68.92% increases compared to the control treatment. At a lower salinity stress level of 2.77 dS m^− 1^, the control group exhibited a mean scion fresh weight of 4.19 g. Treatment with *A. fabrum* led to a mean fresh weight of 5.67 g, indicating 35.28% increases compared to the control. The introduction of 0.4%GB resulted in a significant boost in scion fresh weight, with a mean of 5.00 g, indicating a 19.34% increase over the control. Remarkably, combining 0.4%GB with *A. fabrum* resulted in a mean scion fresh weight of 6.11 g, demonstrating a substantial 45.92% increases compared to the control (Fig. 2a).

#### Rootstock fresh weight

At a salinity stress level of 6.56 dS m^− 1^, the control group displayed a mean rootstock fresh weight of 11.81 g. Treatment with *A. fabrum* resulted in a mean fresh weight of 13.72 g, indicating 16.19% increases compared to the control. Introducing 0.4%GB led to a slightly higher mean fresh weight of 14.01 g, representing an 18.65% increase over the control. However, combining 0.4%GB with *A. fabrum* resulted in a mean rootstock fresh weight of 13.97 g, demonstrating 18.34% increases compared to the control. At a lower salinity stress level of 2.77 dS m^− 1^, the control group exhibited a mean rootstock fresh weight of 14.12 g. Treatment with *A. fabrum* led to a mean fresh weight of 16.62 g, indicating 17.68% increases compared to the control. Introduction of 0.4%GB resulted in a significant boost in rootstock fresh weight, with a mean of 18.52 g, indicating 31.15% increases over the control. Remarkably, combining 0.4%GB with *A. fabrum* resulted in a mean rootstock fresh weight of 19.04 g, demonstrating a substantial 34.85% increases compared to the control (Fig. 2b).

#### Leaf fresh weight

At a salinity stress level of 6.56 dS m^− 1^, the control group displayed a mean leaf fresh weight of 6.81 g. Treatment with *A. fabrum* resulted in a mean fresh weight of 7.40 g, indicating 8.70% increases compared to the control. Introducing 0.4%GB led to a slightly higher mean fresh weight of 7.87 g, representing a 15.50% increase over the control. However, combining 0.4%GB with *A. fabrum* resulted in a mean leaf fresh weight of 8.79 g, demonstrating a substantial 29.06% increases compared to the control. At a lower salinity stress level of 2.77 dS m^− 1^, the control group exhibited a mean leaf fresh weight of 8.17 g. Treatment with *A. fabrum* led to a mean fresh weight of 9.03 g, indicating 10.54% increases compared to the control. Introduction of 0.4%GB resulted in a significant boost in leaf fresh weight, with a mean of 9.94 g, indicating 21.64% increases over the control. Remarkably, combining 0.4%GB with *A. fabrum* resulted in a mean leaf fresh weight of 11.38 g, demonstrating a substantial 39.27% increase compared to the control (Fig. 2c).

#### leaf dry weight

At a salinity stress level of 6.56 dS m^− 1^, the control group displayed a mean leaf dry weight of 2.55 g. Treatment with *A. fabrum* resulted in a mean dry weight of 3.08 g, indicating 20.44% increases compared to the control. Introducing 0.4%GB led to a slightly higher mean dry weight of 3.17 g, representing a 24.06% increase over the control. However, combining 0.4%GB with *A. fabrum* resulted in a mean leaf dry weight of 3.49 g, demonstrating a substantial 36.49% increases compared to the control. At a lower salinity stress level of 2.77 dS m^− 1^, the control group exhibited a mean leaf dry weight of 3.07 g. Treatment with *A. fabrum* led to a mean dry weight of 3.82 g, indicating a 24.24% increase compared to the control. Introduction of 0.4%GB resulted in a significant boost in leaf dry weight, with a mean of 4.78 g, indicating a 55.76% increase over the control. Remarkably, combining 0.4%GB with *A. fabrum* resulted in a mean leaf dry weight of 5.74 g, demonstrating a substantial 86.76% increase compared to the control (Fig. 2d).

#### Root fresh weight

**Fig. 3:**
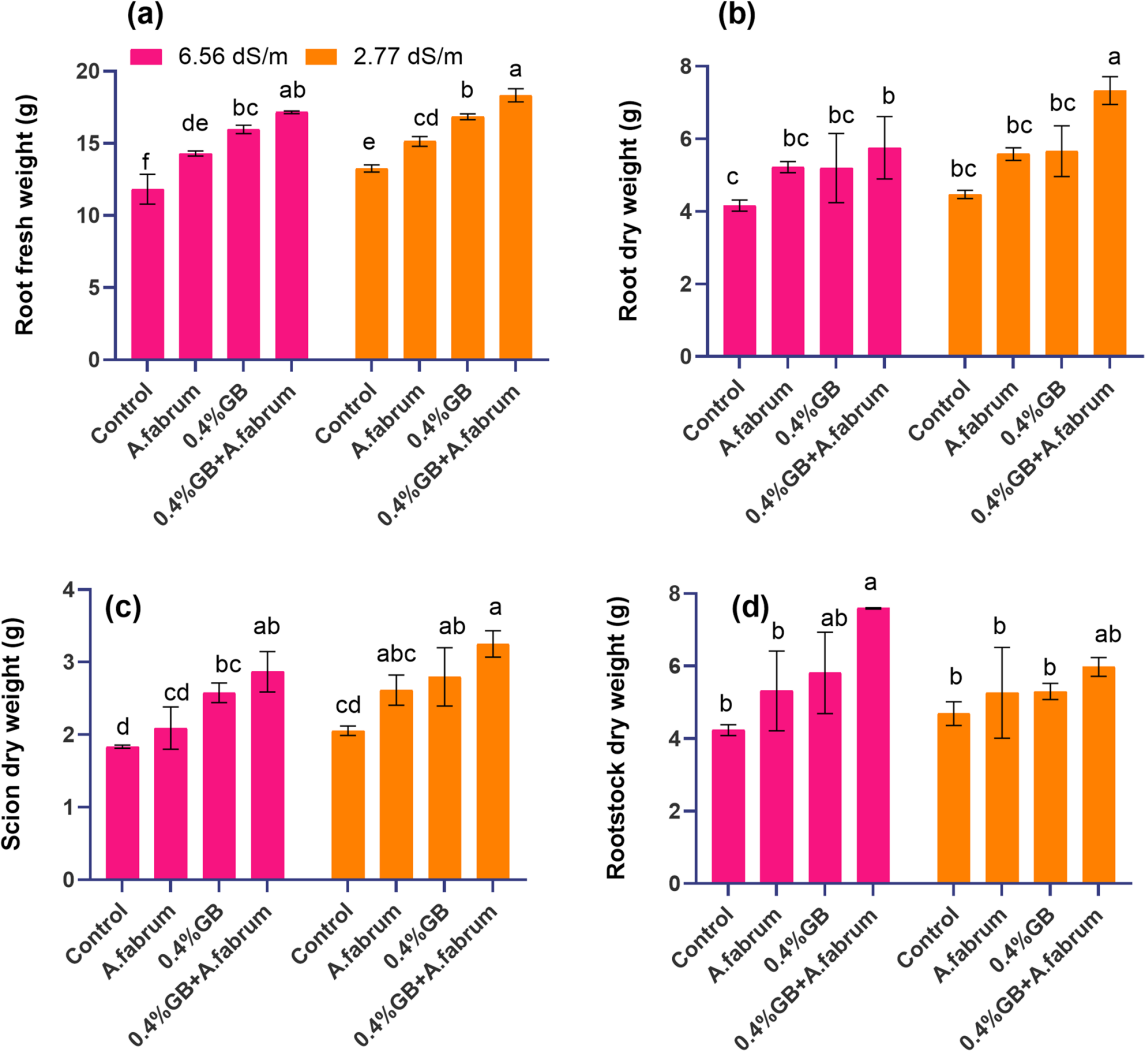
Effect of applied treatments on root fresh weight (**a**), root dry weight (**b**), scion dry weight (**c**) and rootstock dry weight (**d**) of mango seedlings under saline and non-saline growth conditions. The bars are showing the mean (*n* = 3) and different letters are showing the statistical differences based on Tukey’s HSD test among the treatments at *p* ≤ 0.05

At a salinity stress level of 6.56 dS m^− 1^, the control group displayed a mean root fresh weight of 11.82 g. Treatment with *A. fabrum* resulted in a mean fresh weight of 14.30 g, indicating a 20.94% increase compared to the control. Introducing 0.4%GB led to a higher mean fresh weight of 15.97 g, representing a 35.05% increase over the control. Moreover, combining 0.4%GB with *A. fabrum* resulted in the highest mean root fresh weight of 17.15 g, demonstrating a substantial 45.02% increase compared to the control. At a lower salinity stress level of 2.77 dS m^− 1^, the control group exhibited a mean root fresh weight of 13.26 g. Treatment with *A. fabrum* led to a mean fresh weight of 15.14 g, indicating a 14.11% increase compared to the control. Introduction of 0.4%GB resulted in a significant boost in root fresh weight, with a mean of 16.84 g, indicating a 26.94% increase over the control. Remarkably, combining 0.4%GB with *A. fabrum* resulted in a mean root fresh weight of 18.33 g, demonstrating a substantial 38.17% increase compared to the control (Fig. 3a).

#### Root dry weight

At a salinity stress level of 6.56 dS m^− 1^, the control group displayed a mean root dry weight of 4.17 g. Treatment with *A. fabrum* resulted in a mean dry weight of 5.22 g, indicating a 25.30% increase compared to the control. Introducing 0.4%GB led to a similar mean dry weight of 5.20 g, representing a 24.74% increase over the control. Moreover, combining 0.4%GB with *A. fabrum* resulted in the highest mean root dry weight of 5.76 g, demonstrating a substantial 38.14% increase compared to the control. At a lower salinity stress level of 2.77 dS m^− 1^, the control group exhibited a mean root dry weight of 4.47 g. Treatment with *A. fabrum* led to a mean dry weight of 5.58 g, indicating a 24.76% increase compared to the control. Introduction of 0.4%GB resulted in a significant boost in root dry weight, with a mean of 5.66 g, indicating a 26.65% increase over the control. Remarkably, combining 0.4%GB with *A. fabrum* resulted in a mean root dry weight of 7.34 g, demonstrating a substantial 64.01% increase compared to the control (Fig. 3b).

#### Scion dry weight

At a salinity stress level of 6.56 dS m^− 1^, the control group displayed a mean scion dry weight of 1.83 g. Treatment with *A. fabrum* resulted in a mean dry weight of 2.09 g, indicating a 14.02% increase compared to the control. Introducing 0.4%GB led to a higher mean dry weight of 2.58 g, representing a 40.52% increase over the control. Moreover, combining 0.4%GB with *A. fabrum* resulted in a mean scion dry weight of 2.87 g, demonstrating a substantial 56.43% increase compared to the control. At a lower salinity stress level of 2.77 dS m^− 1^, the control group exhibited a mean scion dry weight of 2.05 g. The *A. fabrum* led to mean dry weight of 2.61 g indicating the 27.39% increase and 0.4%GB showed dry weight of 2.80 g, indicating a 36.27% increase compared to the control. Remarkably, combining 0.4%GB with *A. fabrum* resulted in the highest mean scion dry weight of 3.25 g, demonstrating a substantial 58.36% increase compared to the control (Fig. 3c).

##### Rootstock dry weight

At a salinity stress level of 6.56 dS m^− 1^, the control group displayed a mean rootstock dry weight of 4.23 g. Treatment with *A. fabrum* resulted in a mean dry weight of 5.31 g, indicating a 25.56% increase compared to the control. Introducing 0.4%GB led to a higher mean dry weight of 5.81 g, representing a 37.32% increase over the control. Moreover, combining 0.4%GB with *A. fabrum* resulted in the highest mean rootstock dry weight of 7.60 g, demonstrating a substantial 79.47% increase compared to the control. At a lower salinity stress level of 2.77 dS m^− 1^, the control group exhibited a mean rootstock dry weight of 4.69 g. Treatment with *A. fabrum* resulted in a mean dry weight of 5.26 g, indicating a 12.18% increase compared to the control. Introduction of 0.4%GB led to a similar mean dry weight of 5.30 g, representing a 12.97% increase over the control. Remarkably, combining 0.4%GB with *A. fabrum* resulted in a mean rootstock dry weight of 5.98 g, demonstrating a substantial 27.40% increase compared to the control (Fig. 3d).

### Gas exchange parameters

#### Total chlorophyll

**Fig. 4:**
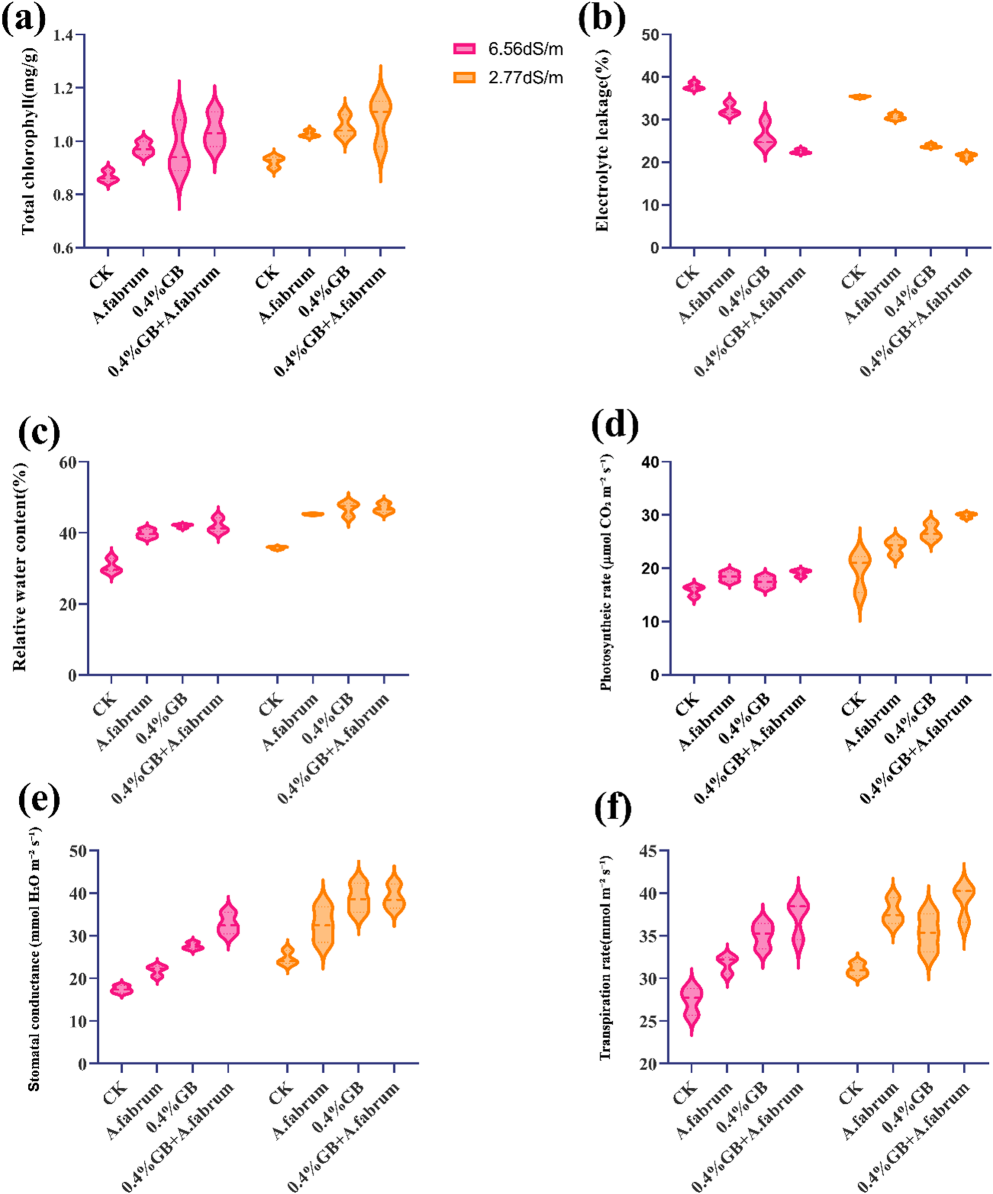
Effect of applied treatments on total chlorophyll (**a**), electrolyte leakage (**b**), relative water content (**c**), photosynthetic rate (**d**), stomatal conductance (**e**) and transpiration rate (**f**) of mango seedlings under saline and non-saline growth conditions

At a salinity stress level of 6.56 dS m^− 1^, the control group displayed a mean total chlorophyll content of 0.87mg g^− 1^. Treatment with *A. fabrum* or 0.4%GB led to a mean chlorophyll content of 0.97 mg g^− 1^ each, indicating a 12.46% and 11.99% increase, respectively compared to the control. Moreover, combining 0.4%GB with *A. fabrum* resulted in the highest mean total chlorophyll content of 1.04 mg g^− 1^, demonstrating a substantial 20.04% increase compared to the control. At a lower salinity stress level of 2.77 dS m^− 1^, the control group exhibited a mean total chlorophyll content of 0.92 mg g^− 1^. Treatment with *A. fabrum* led to a mean chlorophyll content of 1.03 mg g^− 1^, indicating a 10.99% increase compared to the control. Introduction of 0.4%GB resulted in a slightly higher mean chlorophyll content of 1.05 mg g^− 1^, representing a 13.97% increase over the control. Remarkably, combining 0.4%GB with *A. fabrum* resulted in a mean total chlorophyll content of 1.08 mg g^− 1^, demonstrating a substantial 16.86% increase compared to the control (Fig. 4a).

#### Relative water content (RWC)

At a salinity stress level of 6.56 dS m^− 1^, the control group exhibited a mean RWC of 30.52%. Treatment with *A. fabrum* resulted in an increased mean RWC of 39.76%, indicating a 30.27% increase compared to the control. Introducing 0.4%GB led to a further increase in mean RWC to 41.94%, representing a 37.41% increase over the control. Moreover, combining 0.4%GB with *A. fabrum* also resulted in a mean RWC of 41.94%, demonstrating a similar 37.41% increase compared to the control. At a lower salinity stress level of 2.77 dS m^− 1^, the control group displayed RWC of 35.77%. Treatment with *A. fabrum* resulted in an increased mean RWC of 45.22%, indicating a 26.43% increase compared to the control. Introduction of 0.4%GB led to a further increase in mean RWC to 46.82%, representing a 30.91% increase over the control. Remarkably, combining 0.4%GB with *A. fabrum* also resulted in a mean RWC of 46.86%, demonstrating a similar 31.02% increase compared to the control (Fig. 4c).

#### Photosynthetic rate (µmol CO₂ m⁻² s⁻¹)

At a salinity stress level of 6.56 dS m^− 1^, the treatment with *A. fabrum* resulted in an increased photosynthetic rate of 16.46% over to the control. Introducing 0.4%GB led to a slightly lower mean photosynthetic rate representing a 10.14% increase over the control. Moreover, combining 0.4%GB with *A. fabrum* resulted in the highest photosynthetic rate of 20.88% increase compared to the control. At a lower salinity stress level of 2.77 dS m^− 1^, treatment with *A. fabrum* resulted in an increase of 22.87% increase compared to the control. Introduction of 0.4%GB led to a further increase in mean photosynthetic rate of 36.91% over the control. Remarkably, combining 0.4%GB with *A. fabrum* resulted in the highest mean photosynthetic rate of about 52.97% increase compared to the control (Fig. 4d).

#### Stomatal conductance (mmol H₂O m⁻² s⁻¹)

At a salinity stress level of 6.56 dS m^− 1^, treatment with *A. fabrum* resulted in an increased mean stomatal conductance 25.37% increase compared to the control. Introducing 0.4%GB led to a further increase in stomatal conductance to 58.04% increase over the control. Moreover, combining 0.4%GB with *A. fabrum* resulted in the highest stomatal conductance about 88.06% increase compared to the control. At a lower salinity stress level of 2.77 dS m^− 1^, treatment with *A. fabrum* resulted in an increased mean stomatal by 31.82% increase compared to the control. Introduction of 0.4%GB led to a further increase in stomatal conductance to 56.96% increase over the control. Remarkably, combining 0.4%GB with *A. fabrum* resulted in a mean stomatal conductance demonstrating a substantial 57.89% increase compared to the control (Fig. 4e).

#### Transpiration rate (mmol m⁻² s⁻¹)

At a salinity stress level of 6.56 dS m^− 1^, treatment with *A. fabrum* resulted in an increased mean transpiration rate about 15.78% increase compared to the control. Introducing 0.4%GB led to a further increase in mean transpiration rate to 27.92% increase over the control. Moreover, combining 0.4%GB with *A. fabrum* resulted in the highest mean transpiration rate of 35.62% increase compared to the control. At a lower salinity stress level of 2.77 dS m^− 1^, treatment with *A. fabrum* resulted in an increased mean transpiration rate indicating a 21.77% increase compared to the control. Introduction of 0.4%GB led to a slightly lower transpiration rate, representing a 13.91% increase over the control. Remarkably, combining 0.4%GB with *A. fabrum* resulted in a transpiration rate, demonstrating a substantial 25.81% increase compared to the control (Fig. 4f).

### Stress indicator parameters

#### Electrolyte leakage (EL)

**Fig. 5:**
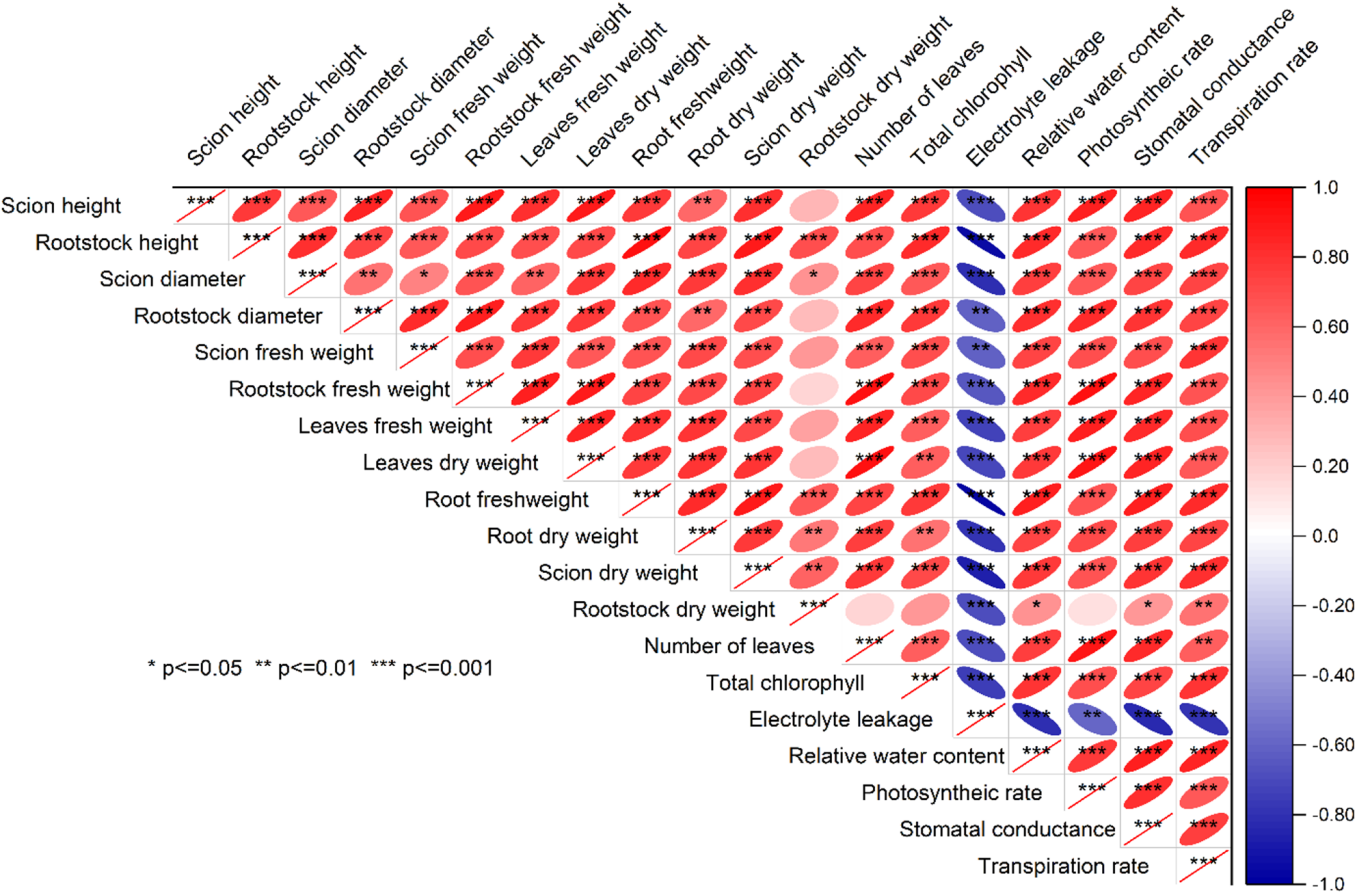
Pearson’s correlation among the studied attributes

At a salinity stress level of 6.56 dS m^− 1^, the control group exhibited a mean EL of 37.85%. Treatment with *A. fabrum* resulted in a decreased mean EL of 32.38%, indicating a decrease of 14.45% change compared to the control. Introducing 0.4%GB led to a further decrease in mean EL to 26.38%, representing a reduction of 30.31% change compared to the control. Moreover, combining 0.4%GB with *A. fabrum* resulted in the lowest EL mean of 22.53%, demonstrating a substantial 40.49% decrease compared to the control. At a lower salinity stress level of 2.77 dS m^− 1^, the control group displayed an EL of 35.35%. Treatment with *A. fabrum* resulted in a decreased.

mean EL of 30.65%, indicating a 13.29% reduction compared to the control. Introduction of 0.4%GB led to a further decrease in mean EL to 23.83%, representing a 32.59% decrease compared to the control. Remarkably, combining 0.4%GB with *A. fabrum* resulted in the lowest mean EL of 21.41%, demonstrating a substantial decrease of 39.44% compared to the control (Fig. 4b) (Fig. 5).

## Discussion

Soil salinization primarily arises from the accumulation of water-soluble salts such as sodium (Na⁺), potassium (K⁺), chloride (Cl⁻), and sulfate (SO₄^−2^) in the root zone. This buildup alters osmotic potential, restricts water uptake by roots, and disturbs plant cellular homeostasis [40–42]. Similar to other abiotic stresses, the severity of growth inhibition under salinity depends on plant species, developmental stage, and salinity levels [43]. At the molecular level, salinity can downregulate essential regulatory genes associated with the cell cycle (e.g., cyclins, CDKs), thereby reducing cell division and overall growth [44]. This reduced growth capacity limits nutrient and water uptake and ultimately suppresses biomass accumulation. Zhu et al. (2022) demonstrated that salt-sensitive plants often exhibit immediate growth cessation under stress, while tolerant genotypes maintain growth through rapid activation of defense responses [45, 46].

Our study confirmed that mango (*Mangifera indica* L.), a salinity-sensitive fruit crop, exhibited reduced growth, biomass, and physiological performance under high salinity levels, consistent with earlier reports [47, 48]. Importantly, both *Agrobacterium fabrum* and glycine betaine (GB) individually improved seedling performance, but the combined treatment produced the most pronounced effects. This suggests a synergistic interaction between microbial and osmoprotectant mechanisms.The enhanced effect of the combined treatment may be explained by the complementary roles of the two agents. *A. fabrum* is known to produce phytohormones (e.g., IAA, cytokinins and ACC deaminase), which, lower ethylene accumulation under stress, thereby promoting root growth and nutrient uptake [18, 20]. In parallel, GB stabilizes membranes, protects photosynthetic machinery, and acts as a ROS scavenger [13–15]. Together, these mechanisms likely strength ion balance, water retention, and photosynthetic capacity, leading to improved growth under salinity. Similar synergistic outcomes of PGPR and osmolytes have been documented in other crops [1, 2]. A critical aspect of salinity tolerance is maintaining Na⁺/K⁺ homeostasis. Excess Na⁺ disrupts cytoplasmic enzyme activity and photosynthesis, whereas a high K⁺/Na⁺ ratio is essential for osmotic adjustment and metabolic stability [35, 49]. Our results suggest that GB contributed to osmotic adjustment, while *A. fabrum* likely improved nutrient uptake and transport efficiency, indirectly supporting a favorable Na⁺/K⁺ balance. This is consistent with recent findings that PGPR regulate ion transporters and maintain ionic homeostasis under saline conditions [2, 3].

The combined treatment also resulted in higher chlorophyll content, photosynthetic rate, and stomatal conductance. These improvements may be attributed to GB-mediated stabilization of thylakoid membranes and osmotic protection, alongside, *A. fabrum*-mediated enhancement of nutrient availability and hormonal regulation. Lower electrolyte leakage under combined treatment further confirmed improved membrane stability and reduced oxidative damage.

Our results align with reports showing that GB protects photosynthesis by modifying lipid composition of thylakoid membranes in wheat [50], while also increasing soluble sugar and amino acid accumulation to maintain osmotic balance in legumes [51]. Similarly, *A. fabrum* has been reported to enhance drought and salinity tolerance in cereals by improving root architecture and antioxidant activity [52, 53]. The observed improvements in mango seedlings corroborate findings from other PGPR–GB studies, such as improved yield and stress resilience in tomato and rice [1, 4]. However, not all studies report strong synergism. In some cases, PGPR alone was sufficient to induce salt tolerance, while GB addition had limited benefits [3]. These contrasting findings highlight that the magnitude of synergy depends on species, developmental stage, and stress intensity. Our results suggest that in mango seedlings, which are highly salt-sensitive, the dual application is particularly effective. While the present study demonstrates promising outcomes, further work is needed to elucidate the molecular mechanisms underlying the synergy [54, 55]. Future research should investigate stress-related gene expression (e.g., SOS pathway for Na⁺ extrusion, antioxidant enzyme genes), hormonal cross-talk, and long-term effects on fruit yield [56]. Integrating physiological and transcriptomic analyses would help clarify how PGPR–GB interactions regulate stress signaling pathways in mango.

## Conclusion

This study demonstrated that the combined application of *Agrobacterium fabrum* (ATCC 23308) and glycine betaine significantly improved the growth, biomass, chlorophyll content, relative water content, and gas-exchange parameters of mango seedlings under salinity stress. The treatments also reduced electrolyte leakage, indicating enhanced membrane stability and stress tolerance. While each treatment alone provided measurable benefits, the combined application proved most effective, underscoring a synergistic interaction between microbial-mediated nutrient uptake and hormonal regulation, and GB-mediated osmoprotection. To our knowledge, this is the first report on the integrative use of *A. fabrum* and glycine betaine for enhancing salinity tolerance in mango seedlings. These findings provide a novel and sustainable strategy for managing salinity stress in mango cultivation, with potential applications for improving fruit production in salt-affected regions [57–68].

## Data Availability

All the data generated or analyzed during this study are included in this submitted manuscript.
